# Detecting Latent Topics and Trends in Global Publications on Brucellosis Disease Using Text Mining

**DOI:** 10.1155/2022/7274734

**Published:** 2022-03-03

**Authors:** Meisam Dastani, Jalal Mardaneh, Omid Pouresmaeil

**Affiliations:** ^1^Infectious Diseases Research Center, Gonabad University of Medical Sciences, Gonabad, Iran; ^2^Department of Microbiology, School of Medicine, Infectious Diseases Research Center, Gonabad University of Medical Sciences, Gonabad, Iran; ^3^Department of Microbiology and Virology, Faculty of Medicine, Mashhad University of Medical Sciences, Mashhad, Iran

## Abstract

**Purpose:**

Brucellosis is widespread globally and one of the most important zoonotic diseases. Therefore, to fully comprehend the disease and discover ways of prevention and treatment, researchers have conducted some research in this field. Hence, this study will focus on the topic trend of scientific publications of brucellosis.

**Methods:**

This study is an applied research using text mining techniques with an analytical approach. The statistical population of the present research is all global publications related to brucellosis. For data extraction, the Scopus citation database was used in the period from 1900 to 2020. The main keywords for search strategy design have been extracted from consultation with thematic specialists and using MESH. Python programming language has been applied to analyze data and implement text mining algorithms.

**Results:**

According to results, eight main topics of “Prevention,” “Clinical symptoms,” “Diagnosis,” “Control,” “Treatment,” “Immunology,” “Structural Features,” and “Pathogenicity” have been identified for brucellosis publications. Moreover, the topics “Prevention” and “Pathogenicity” had the highest and lowest prevalence in the field of brucellosis over time, respectively.

**Conclusion:**

This study has revealed the topics published in the global publications of brucellosis; the findings can be useful for research centers and universities in determining research priorities in the field of brucellosis.

## 1. Introduction

Brucellosis is one of the zoonotic infections between humans and animals, known as undulant fever in humans and brucellosis in animals. The disease is globally widespread due to the dissemination of the infection among domestic and wild animals [1]. The consumption of contaminated fresh milk is the most common way of transmission of brucellosis in endemic countries [2]. Moreover, in different countries, brucellosis is transmitted to humans through the consumption of raw dairy products, especially unpasteurized and contaminated milk and cheese, colostrum, contaminated meat, and contact with the secretions of infected animals [3]. Undulant fever is an occupational hazard for farmers, ranchers, veterinarians, and slaughterhouse workers [4]. Brucellosis is an important disease globally, especially in the Persian Gulf, the Mediterranean, North and East Africa, the Middle East, the Indian subcontinent, Central and South Asia, South and Central America, and parts of Mexico [5].

Due to the importance of the disease, researchers have conducted many studies in this field and published their findings to reach a broad understanding of the disease and discover methods of prevention and treatment. In recent years, due to the increasing rate of online article submission in the information databases apace with the annual publication trend of research, manual review of this research is impossible or extremely time-consuming [6]. Therefore, there is an increasing interest in considering the application of intelligent data extraction methods and strategies from articles in the field of biomedical sciences.

Investigating the publication trend of articles in various scientific fields can provide a proper insight into the efforts of researchers in various fields of knowledge, especially in life sciences. Reviewing the published articles in the field of biomedical sciences makes it possible to identify alterations in the field of knowledge, intelligent classification of information, and identification of different subject areas of knowledge. Therefore, it is necessary to use the methods and techniques of automatic and intelligent investigation of scientific publications.

Furthermore, investigating the publication trend of each scientific field in a specific period represents how much its main issues and subtopics have changed over this time and what subfields each scientific field includes. Moreover, the review of research topics in each scientific field indicates that the highest and lowest research popularities during different periods are assigned to what topics [7]; these results are useful in scientific and research policy-making and prioritization of research problems based on the needs of the researchers.

In this regard, text mining is one of the techniques capable of intelligent data and knowledge extraction from a large volume of scientific publications in various fields of medical sciences, and its purpose is to extract hidden knowledge from texts [8]. As one of the content analysis methods, text mining draws a conceptual model. It visualizes the information through processing, extraction, and sorting of information, as well as enables the analysis, navigation, representation, and disclosure of knowledge structure [9]. Topic modeling is also one of the text mining algorithms applied to discover the topics of scientific publications and identify the publication trend of topics over a period of time [10]. Moreover, this algorithm can detect trendy topics and subjects that have been less discussed by researchers [7].

In this regard, various studies in different fields of medical sciences have applied text mining and topic modeling techniques to discover hidden knowledge and identify topics in scientific publications, for example, studies in the subject areas of anesthesia [11], drug safety [12], coronavirus [10], COVID-19 [13–15], and urothelial cancer [16].

Accordingly, the analysis of texts published in citation databases and identifying research trends in various scientific fields is one of the most important applications of text mining. Therefore, discovering and analyzing the structure of topics and identifying the topic evolution of scientific publications of brucellosis over a specific time are the main issues addressed in the present investigation.

## 2. Materials and Methods

### 2.1. Data Set and Data Collection

This study is applied research that has been carried out by applying text mining techniques and an analytical approach. The statistical population includes all scientific publications of the subject area of brucellosis in the Scopus citation database, which is considered a suitable database for scientometric research due to its comprehensiveness for various fields of science and indexing a significant number of articles [17–19]. Medical Subject Headings (MESH) database was used, and it was consulted with microbiology experts to determine the main keywords in search strategy design. Then, the brucellosis publications were extracted in the advanced search section of the Scopus database using the following search strategy on June 2, 2021; the time period considered in the search strategy was from 1900 to 2020, and data were extracted in CSV file format.

(TITLE (brucellosis) OR TITLE (malta AND fever) OR TITLE (gibraltar AND fever) OR TITLE (rock AND fever) OR TITLE (cyprus AND fever) OR TITLE (brucella AND infection) OR TITLE (brucella AND infections) OR TITLE (undulant AND fever) OR TITLE (brucellosis) OR TITLE (bangs AND disease) OR TITLE (bang AND disease) OR TITLE-ABS-KEY (brucella) OR TITLE (pulmonary AND brucellosis) OR TITLE (mediterranean AND fever)).

### 2.2. Preprocessing

Titles, abstracts, and keywords of retrieved publications were merged to perform the text mining process. Afterward, preprocessing and data cleansing operations were applied to the studied data for increasing data quality and validity of patterns and extracted relationships. It is noteworthy that data cleansing only maintains the required and relevant textual data [20].

The following steps were implemented to perform data cleansing operations:

- Eliminating unimportant graphemes, including extra empty spaces, text formatting tags, and removing nonalphabetic graphemes such as deleting punctuation marks or numbers from the text; these operations lead to focusing on the words and phrases in the text [21].
- Tokenization of the text components into words and phrases, which is one of the essential processes of text preprocessing in text mining [22].
- Converting capital letters to lowercase letters for the purpose of text uniformity; since capital and lowercase words are stored in a separate and different computer language, converting all capitals to lowercase letters makes all words uniform [23].
- The uniforming of synonymous words was performed manually. The lemmatization method was also applied to make different forms of uniform words. Lemmatization is replacing words or their basic dictionary forms with the conjugated forms of words [24]. Lemmatization also considers a part of the roles of words in the text and uses the dictionary of words to convert the basic form of words. This method has been proposed for word uniforming in topic modeling [21, 25].
- Removing stop words and some words that are not valuable for retrieving or analyzing documents [21], such as conjunctions and suffixes (and, the, of, and for) that have low-value information content. Moreover, by evaluating the textual data, other words that were repeated in the text and did not have a particular meaning in the text were added to the list of stop words and removed from the text.

### 2.3. Topic Modeling

Topic modeling is a statistical and textual analysis method that evaluates documents to identify their themes or topics. The results of this algorithm can be used to analyze how topics relate to each other and how they evolve over time [26]. Topic models are based on the idea that documents consist of a set of topics, where a topic is defined as a probability distribution on the words [27].

In the present research, a topic modeling algorithm named the latent Dirichlet allocation (LDA) has been applied for topic modeling operations. LDA is one of the most important methods of implementing topic modeling and one of the best and most extensively used algorithms, which significantly identifies relevant semantic issues in scientific texts [26].

Latent Dirichlet allocation (LDA) is a probabilistic model that can extract latent topics from a collection of documents. According to Blei et al., topic modeling assumes that each document of a corpus is structured by a set of hidden topics [28]. By applying this method, researchers can uncover the latent topics and find relationships between topics from the textual data [28].

Many social scientists have used topic modeling, especially latent Dirichlet allocation (LDA), which is the most popular method of topic modeling [10, 16, 29, 30].

Since the LDA method cannot determine the number of appropriate topics, the coefficient of variation (Cv) coherence measure was applied. An important index, called Cv coherence, is employed for measuring the co-occurrence of the words extracted using the topic model. The model's great performance is interpreted if the words from similar topics co-occur frequently, otherwise stated, in the case of high Cv coherence [31].

It was indicated in previous studies that the performance of this criterion is excellent in determining the number of topics and is closely related to human judgments about the interpretation of topics [32, 33].

In this article, at the outset, the number of main topics was obtained for all data of brucellosis publications, and then, the determination of the number of topics and topic modeling was performed for publications related to each of the obtained topics; hence, the subtopics dependent on each main topic were also identified.

Furthermore, after the words were identified, the publications related to each topic were labeled for that topic by consultation with a topical expert.

Python programming language and text mining-related libraries, including Gensim, NLTK, and SpaCy, have been applied to implement these steps [34]. Python is an open-source, compact, and versatile programming language that possesses simple syntax; its development is straightforward and provides users with various libraries for working with texts [34].

## 3. Results

**Figure 1:**
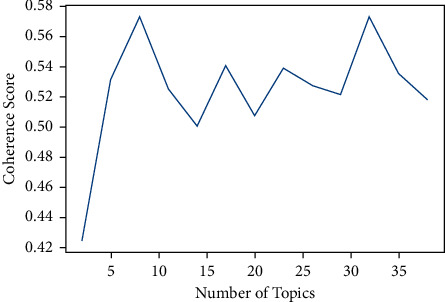
Value of Cv coherence in different numbers of topics.

A total of 25,846 publications related to brucellosis were extracted from the Scopus database. The LDA topic modeling has been used to select the number of topics.  Figure 1 indicates the value of Cv coherence in different numbers of topics.

Figure 1 shows the values of Cv coherence for the number of topics in the range of 2–40 subjects; thus, the highest value of Cv coherence is related to eight topics, and therefore, eight subjects were selected for topic modeling of brucellosis publications.

**Figure 2:**
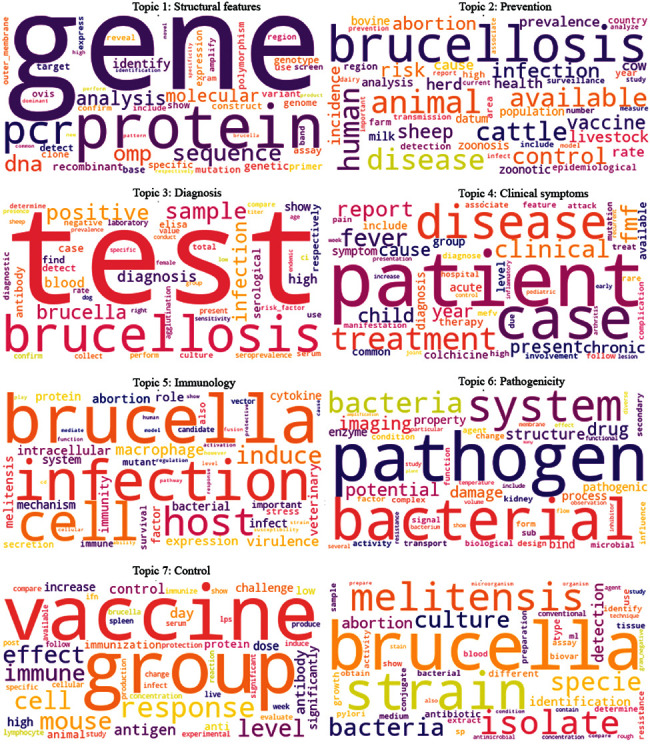
Word cloud of the eight topics of brucellosis scientific publications.

The results obtained from topic modeling are shown in the word cloud presented in Figure 2. The topic label is also specified according to the words specified in each topic.

Figure 2 indicates the ten most important words of each of the eight topics in the word cloud format. In the word cloud, the words are more important and useful and have larger fonts in that topic. Moreover, in each word cloud, the purpose of the different colors of words is to separate the words from each other.

**Figure 3:**
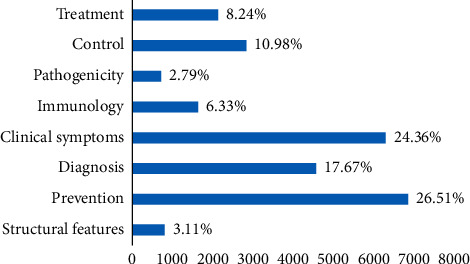
Publication rates of each of the brucellosis publications.

Furthermore, Figure 3 illustrates the diagram of the publication rates in each topic.

Figure 3 indicates the publication rates in each of the topics. According to Figure 3, “Prevention” with 26.31% and “Pathogenicity” with 2.79% have the highest and lowest publication rates in the field of brucellosis, respectively.  Table 1 shows the subtopics of each of the eight topics, along with 10 of the most important and relevant words in each subtopic.

**Figure 4:**
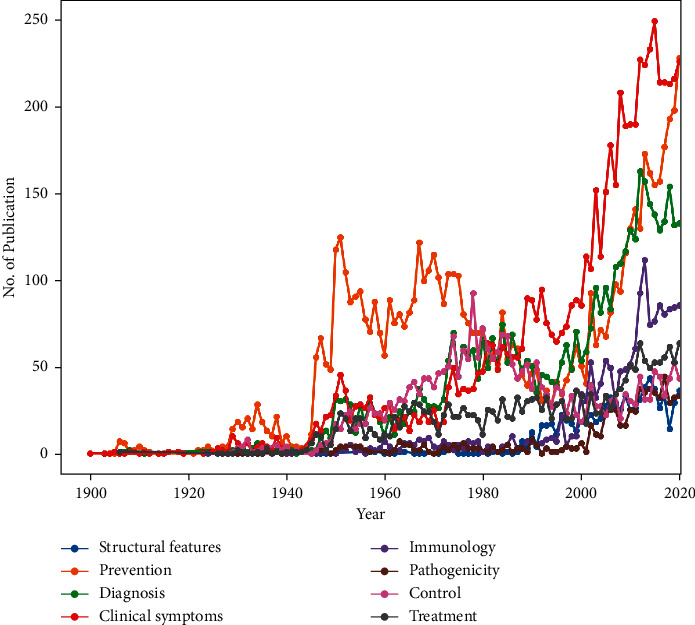
Publication trend of brucellosis publications over a period of time.

It is indicated in Table 1 that the topics “Structural features,” “Prevention,” “Diagnosis,” “Clinical symptoms,” “Immunology,” “Pathogenicity,” and “Treatment” had 5, 5, 8, 8, 11, 4, and 4 subtopics, respectively.  Figure 4 also shows the publication rate of each of the subtopics.  Figure 4 indicates the publication rate of each subtopic.

Table 1 also indicates that in the topic “structure features,” the subtopic “protein” has the highest publication rate with 36.32% and the subtopic “omp” has the lowest publication rate with 4.85%.

In the topic “Prevention,” the highest publication rate is dedicated to the subtopic “Vaccine” with 49.96%, and the subtopic “Bioterrorism” has the lowest publication rate with 2.80%.

In the topic “Diagnosis,” the highest publication rate belongs to the subtopic “Elisa” with 33.70%, and the lowest publication rate belongs to the subtopic “Brucellacapt” with 1.40%.

In the topic “Clinical symptoms,” the subtopics “disease” with 25.25% and “Fever” with 1.24% have the highest and lowest publication rates, respectively.

In the topic “Immunology,” the subtopic “macrophage” with 35.98% has the highest publication rate, and the subtopic “lysosomal” with 0.12% has the lowest publication rate.

In the topic “Pathogenicity,” the subtopics “pathogens” with 63.33% and “*Brucella*” with 36.67% have the highest and lowest publication rates, respectively.

In the topic “control,” the subtopic “vaccine” with 51.66% and the subtopic “patient” with 48.34% have the highest and lowest publications rates, respectively.

In the topic “treatment,” the subtopic “melitensis” with 51.95% has the highest publication rate, and the subtopic “Bacteria” with 8.59% has the lowest publication rate.

Figure 4 indicates the publication trends of the eight topics of scientific publications of brucellosis.

According to Figure 4, the “Clinical Symptom” has been the most frequently published topic since 1990, and “Prevention” has been the most widely published from 1945 to 1980, although this topic has had the highest publication rate in recent years after the topic “Clinical Symptoms.” It also had the highest number of publications after “Clinical Symptom.” The topics “Diagnosis” and “Immunology” have been in the next ranks in terms of publications rate in recent years. Other topics have also had an approximately constant publication trend over time, although these topics have also been on the rise since 2000.

## 4. Discussion

*Brucella* is a Gram-negative, optional intracellular, nonmotile, non-spore-forming coccobacillus causing brucellosis [35]. Undulant fever is a systemic and progressive disease that is considered a serious health problem [36]. Human brucellosis has been largely controlled in many countries in recent decades due to improvements in health and economy in those societies. By all means, international exchanges of livestock products and the migration and traveling of people around the world have affected the epidemiological situation of brucellosis. However, many cases of brucellosis are still reported annually in the world and are considered a health challenge, especially in areas where human brucellosis infections are common [35, 37]. Accordingly, researchers are conducting various studies to respond to this disease. Bakri claimed that the field of brucellosis is highly dynamic due to the high publication rate of research in this field in recent years [38]. Therefore, it is essential to study the publication trends of scientific publications of brucellosis and extract knowledge from this scientific field with text mining and topic modeling techniques.

According to the results of this study, eight main topics have been identified for the scientific publications of brucellosis by investigating the scientific publications of brucellosis. “Prevention,” “Clinical symptoms,” “Diagnosis,” “Control,” “Treatment,” “Immunology,” “Structural features,” and “Pathogenicity” are in the respective order in terms of highest to lowest publication rates for these eight main topics.

The topic “prevention” has the highest publication rate in the field of brucellosis. This topic includes the subtopics “Milk,” “zoonotic,” “livestock,” “bioterrorism,” and “vaccine.” It was also found that most publications on this topic have been dedicated to the subtopic “Vaccine,” which indicates the important role of vaccine use in preventing brucellosis. It also means that research in the field of “vaccine” is in a progressive trend, and today's human need research in the field of brucellosis is focused on the vaccines. Nowadays, there are several types of vaccines available; some of them are suitable for human use and some others for animals. These vaccines are in various types, including “Live,” “Inactive cell lysate,” “Subunit,” “DNA,” “Synthetic peptide,” and “Live vectored” [30].

“Clinical symptoms” was the second subject area of publications with high frequency, and “attack,” “child,” “lesion,” “disease,” acute, “fever,” “amyloidosis,” and “chronic” were its subtopics. The results demonstrated that the subtopic “Disease” had the most application in publications in this field. The children's involvement is also a major challenge in the field of health and, in this regard, has dedicated a large part of the literature on the topic “clinical symptoms.” The disease is known as the “Disease of mistakes” due to its nonspecific clinical symptoms. It was shown in extensive studies in this field that fever is the most obvious clinical sign of the disease and also the highest rate of infection in children and adults occurs in males, the reason of which is that men and boys, compared with women and girls, are more responsible for doing affairs related to livestock such as shepherding and milking [39].

The subject area “Diagnosis” is in the third rank of publication rate of the field of brucellosis, which includes the subtopics “Brucellacapt,” “ELISA,” “detect,” “organism,” “positive,” “PCR,” “culture,” and “laboratory.” Rapid and precise diagnosis of brucellosis is important due to the multisystem involvement in the body. Culture and microbiological identification, serological tests including rose Bengal slide agglutination test, serum agglutination test (SAT), microagglutination test [40], indirect Coombs (antihuman globulin) test, brucellacapt, enzyme-linked immunosorbent assay (ELISA), indirect fluorescent antibody test (IFA), and immunochromatographic lateral flow assay, and some molecular tests such as conventional polymerase chain reaction (PCR) and real-time PCR (RT-PCR) are among the methods applied for the diagnosis of the disease. Serological diagnostic methods are extensively available as commercial kits and can be used for initial screening and final diagnostic confirmation. Meanwhile, ELISA is an appropriate technique with excellent performance for detecting specific IgG subclasses of *Brucella* and other immunoglobulins [41]. According to results, the highest publication rate of the topic “Diagnosis” belongs to “ELISA.”

“Control” is another topic in brucellosis publications, which included the subtopics “Patient” and “vaccine,” and the “vaccine” has been the most studied subtopic in this topic. The term “vaccine” in the “prevention” set is also considered the most common topic in the field, which indicates its significance.

“Treatment” is the next topic in brucellosis publications, which included the subtopics “Culture,” “bacteria,” “*melitensis*,” and “antibiotic.” In this topic, the highest number of phrases has been dedicated to “*melitensis*.” Since *Brucella melitensis* has been the most aggressive among *Brucella* strains and constitutes the most human infections [42], it is obvious that the most attention on the field of treatment of brucellosis has belonged to this strain.

“Immunology” is the subsequent topic in brucellosis publications, which included the subtopics “Intracellular,” “expression,” “macrophage,” “pathway,” “caspase,” “neutrophil,” “response,” “nitric_oxide,” “apoptosis,” “lysosomal,” and “cytokine.” Since the pathogenesis of *Brucella* in the human body depends on its viability and proliferation in macrophage cells, the term macrophage has been the most investigated topic in the field of immunology. “Superoxide dismutase,” “Type IV Secretion System (T4SS),” “Cyclic *β*-1-2-glucans (C*β*G),” “Cytochrome oxidase,” “Nitric oxide reductase (NorD),” and “Brucella virulence factor A (BvfA)” are among the factors of *Brucella* virulence to fight against cells of the human immune system, especially macrophages [43]. The next topic is the “Structural features,” which included the subtopics “Gene,” “omp,” “marker,” “protein,” and “genome.”

The results also revealed that the topic “protein” has the highest publication rate in articles related to the subject area “Structural features.” Moreover, “Pathogenicity” is the topic with the lowest publication rate in the field of brucellosis, which included the subtopics “*Brucella*” and “pathogen,” and the term pathogen has been the most considered term in this field.

In general, the high publication rates in the topics “Prevention,” “Clinical symptoms,” “Diagnosis,” and “Control” can be related to the global publication of articles, easier and faster access to research resources of basic medical sciences, modern scientific tools for studying these topics, and finally nowadays humans' need to prevention, recognition of clinical symptoms, and diagnosis and control of brucellosis. Besides, the low publication rates of the topics “Pathogenicity,” “Structural features,” and “Immunology” can indicate that the research life of these topics with current science and scientific tools is now coming to an end, and a complete scientific knowledge has been developed in these areas. If a new scientific research method or tool is discovered or invented in the world of experimental science, there would be an advancement possibility in scientific research and publications in these areas.

In this context, similar studies tried to identify the topics of scientific publications of other microbial diseases. Danesh et al. have identified eight topics for the global spreading of coronavirus as follows: “structure and proteomics,” “Cell signaling and immune response,” “clinical presentation and detection,” “Gene sequence and genomics,” “Diagnosis tests,” “vaccine and immune response and outbreak,” “Epidemiology and Transmission,” and “gastrointestinal tissue” [10]. Dastani and Danesh have noted topics related to “Prevention,” “Treatment,” and “Diagnosis,” the most important thematic categories in COVID-19 publications of Iranian researchers. It was shown in the study that “symptoms” is the most important subtopic in the “Diagnosis” thematic category [44]. “Global Epidemic Control,” “Hospital Epidemic Control,” “Clinical Treatment,” “Clinical Diagnosis,” “Coronavirus Genetic Components,” “Coronavirus Protein Components and Antibodies,” and “Protease/Proteinase Inhibitors and Viral Inhibiting Drugs” are the topics about SARS scientific publications mentioned by Kostoff and Morse [45]. Nafade et al. have noted “Epidemiology,” “Fundamental research,” “Diagnostics,” “Treatment,” “Vaccines,” and “Operational and PH research” for scientific publications of “tuberculosis” between 2007 and 2016 [46]. The results of this study are consistent with the studies mentioned above and show the structure of thematic knowledge in a scientific field.

Scientific research in the field of clinical microbiology, in chronological order, and in terms of the evolution of scientific research primarily focuses on recognizing the nature of the pathogenic agent, clinical symptoms, diagnosis and treatment, and finally control and prevention of the pathogenic agent, which means that without recognizing the structural features of a pathogenic agent or its pathogenesis, the logical trend of scientific research will never be to seek treatment and control the pathogenic agent. In other words, each of the mentioned eight topical phrases has a specific research life, and the researchers' focus on the subject areas will change with increasing the scientific research on the subject area of brucellosis. However, some factors such as today's scientific need, advances in specific technologies (e.g., molecular science, microscopy, information technology, interdisciplinary sciences, and the emergence of new scientific tools), and researchers' hidden purposes (fast extraction of articles, economic profitability, reputation, other personal aims) affect the logical trend of scientific research. According to the results of the present investigation, the publication of basic topics in the field of brucellosis has been on the rise with a constant logical trend, which can be justified by the advancement of technology and scientific tools. Moreover, some factors, such as the incidence of a pandemic, can also lead to a specific subject area being of high interest. In this regard, Danesh et al. have indicated that the publication trend in the field of coronavirus has been associated with a significant growth by the incidence and prevalence of each of MERS, SARS, and COVID-19 [10].

## 5. Conclusion

The findings of this scientific investigation, as the first text mining study in the field of brucellosis publications, have shown the subject trend of brucellosis publications of the last 120 years. The text mining and investigation of subject areas of scientific topics in clinical microbiology assist in understanding the subject areas and today's scientific need of the world, planning and policy-making for the scientific activity, and publication of articles on the topics related to clinical microbiology. The results of this study are of great importance for the publication databases of scientific articles and can be significantly effective in their future orientation in publishing articles related to brucellosis. These results can also be used by the research centers and universities for the purpose of replanning the proposals related to brucellosis. For future research, it is suggested that the publication trends of global articles and each country's domestic publications are studied and compared, which would be important in the collaboration of global research centers to meet today's scientific need in the field of brucellosis disease.

## Figures and Tables

**Table 1:** Subtopics obtained for each of the main topics of the brucellosis publications.

Topics	Most relevant keywords	Label	Frequency (percentage)
Structural features	Gene, brucellosis, polymorphism, genotype, patient, isolate, control, genetic, strain, susceptibility	Gene	107 (13.31%)
	Gene, brucella, omp, specie, usage, nucleotide, factor, pattern, strain, codon	Omp	39 (4.85%)
	Brucella, assay, pcr, detection, gene, molecular, use, marker, sequence, specific	Marker	158 (19.65%)
	Protein, brucella, omp, gene, melitensis, expression, recombinant, vaccine, cell, clone	Protein	292 (36.32%)
	Gene, genome, sequence, analysis, identify, mutation, available, pathogen, genomic, bacterial	Genome	208 (25.87)
Overall		804 (100%)	
Prevention	Brucella, test, sample, cattle, abortion, herd, melitensis, milk, strain, cow	Milk	670 (9.78%)
	Disease, infection, cause, pathogen, brucella, animal, zoonotic, zoonosis, include, spe	Zoonotic	949 (13.85%)
	Brucellosis, case, human, animal, control, high, disease, rate, incidence, livestock	Livestock	1618 (23.61%)
	Property, biological, detection, laboratory, gene, agent, sequence, error, diagnosis, bioterrorism	Bioterrorism	192 (2.80%)
	Available, brucellosis, vaccine, human, diagnosis, treatment, clinical, immunity, infection, chronic	Vaccine	3424 (49.96%)
Overall		6853 (100%)	
Diagnosis	Die, ci, surveillance, por, brucellacapt, slaughterhouse, science, sat, al, unknown	Brucellacapt	64 (1.40%)
	Test, brucellosis, diagnosis, elisa, serological, antibody, brucella, positive, sensitivity, serum	Elisa	1539 (33.70%)
	Dog, antibody, brucella, canis, infection, pathogen, detect, und, pig, breed	Detect	295 (6.46%)
	Infection, day, infect, strain, available, camel, month, organism, brucella, experimental	Organism	143 (3.13%)
	Brucellosis, test, animal, sample, positive, prevalence, cattle, high, seroprevalence, brucella	Positive	899 (19.68%)
	Sample, brucella, pcr, melitensis, positive, milk, isolate, isolation, blood, molecular	Pcr	351 (7.63%)
	Patient, brucellosis, case, blood, culture, diagnosis, brucella, clinical, positive, treatment	Culture	760 (16.64%)
	Brucellosis, case, infection, disease, fever, human, laboratory, cause, report, available	Laboratory	516 (11.30%)
Overall		4567 (100%)	
Clinical symptoms	Colchicine, treatment, patient, therapy, fmf, attack, drug, month, treat, anti	Attack	355 (5.64%)
	Brucellosis, patient, treatment, case, clinical, brucella, infection, diagnosis, year, child	Child	1452 (23.06%)
	Lesion, cell, skin, inflammatory, immune, pathogenesis, inflammation, tissue, disease, syndrome	Lesion	90 (1.43%)
	Case, report, brucellosis, present, cause, disease, diagnosis, involvement, brucella, rare	Disease	1590 (25.25%)
	Patient, group, level, control, fmf, high, significantly, compare, acute, chronic	Acute	638 (10.13%)
	Fever, woman, variant, msf, unknown_origin, pregnancy, mother, ocular, death, fuo	Fever	78 (1.24%)
	Patient, fmf, disease, mutation, mefv, clinical, fever, child, gene, amyloidosis	Amyloidosis	1224 (19.44%)
	Available, brucellosis, case, fever, chronic, etiology, child, comment, nea_familiar, fiebre_mediterr	Chronic	869 (13.80%)
Overall		6296 (100%)	
Immunology	Host, pathogen, intracellular, cell, bacterial, bacteria, system, interaction, infection, mechanism	Intracellular	174 (10.63%)
	Expression, gene, regulate, regulation, target, transcription, analysis, gntr, system, virb	Expression	33 (2.02%)
	Brucella, strain, virulence, gene, mutant, melitensis, survival, abortion, macrophage, brucellosis	Macrophage	589 (35.98%)
	Protein, response, role, brucella, cell, stress, pathway, play, bacterial, important	Pathway	96 (5.86%)
	Mouse, abortion, infection, dependent, secretion, spleen, caspase, production, demonstrate, infect	Caspase	93 (5.68%)
	Available, patient, peptide, study, iron, neutrophil, fmf, overall, disease, drug	Neutrophil	53 (3.24%)
	Vaccine, brucella, infection, brucellosis, cell, response, induce, immune, melitensis, omp	Response	258 (15.76%)
	Nitric_oxide, tumor, complementation, acid, reactive_oxygen, growth, catalyze, respiratory, vector, peritoneal	Nitric_oxide	3 (0.18%)
	Cell, macrophage, brucella, infection, infect, abortion, induce, intracellular, apoptosis, expression	Apoptosis	309 (18.88%)
	Phagosome, cathepsin, cross, particle, morphological, coat, barrier, lysosomal, intact, er_derive	Lysosomal	2 (0.12%)
	Cytokine, rev, control, stimulate, sulglycotide, pylori, induction, production, significantly, dose	Cytokine	27 (1.65%)
Overall		1637 (100%)	
Pathogenicity	Available, bacterial, brucella, community, treatment, detection, high, activity, effect, bacteria	Brucella	264 (36.67%)
	Protein, bacterial, brucella, pathogen, bacteria, bind, show, enzyme, system, gene	Pathogen	456 (63.33%)
Overall		720 (100%)	
Control	Cell, antibody, response, antigen, effect, blood, lymphocyte, level, immune, patient	Patient	1372 (48.34%)
	Vaccine, brucella, group, brucellosis, mouse, response, infection, animal, immune, strain	Vaccine	1466 (51.66%)
Overall		2838 (100%)	
Treatment	Culture, medium, growth, pylori, sample, blood, bacteria, brucella, condition, system	Culture	523 (24.54%)
	Extract, activity, die, und, group, pdt, bacteria, study, catalase, fly	Bacteria	183 (8.59%)
	Brucella, strain, melitensis, isolate, brucellosis, specie, available, abortion, identification, gene	Melitensis	1107 (51.95%)
	Antibiotic, isolate, bacteria, strain, resistance, patient, activity, test, treatment, blood	Antibiotic	318 (14.92%)
Overall		2131 (100%)	

## Data Availability

The data that support the findings of this study are available from the corresponding author upon reasonable request.
